# Obstructive Sleep Apnoea Syndrome and Weight Loss: Review

**DOI:** 10.1155/2012/163296

**Published:** 2012-01-23

**Authors:** Douglas C. Cowan, Eric Livingston

**Affiliations:** ^1^Sleep Department, Gartnavel General Hospital, Glasgow G12 0YN, UK; ^2^Respiratory Department, Glasgow Royal Infirmary, 16 Alexandra Parade, Glasgow G31 2ER, UK

## Abstract

Obstructive sleep apnoea (OSA) syndrome is common, and obesity is a major risk factor. Increased peripharyngeal and central adiposity result in increased pharyngeal collapsibility, through increased mechanical loading around the upper airway, reduced tracheal traction on the pharynx, and reduced neuromuscular activity, particularly during sleep. Significant and sustained weight loss, if achieved, is likely to be a useful therapeutic option in the management of OSA and may be attempted by behavioural, pharmacological, and surgical approaches. Behavioural therapy programs that focus on aspects such as dietary intervention, exercise prescription patients and general lifestyle counselling have been tested. Bariatric surgery is an option in the severely obese when nonsurgical measures have failed, and laparoscopic adjustable gastric banding and Roux-en-Y gastric bypass are the most commonly employed techniques in the United Kingdom. Most evidence for efficacy of surgery comes from cohort studies. The role of sibutramine in OSA in the obese patients has been investigated, however, there are concerns regarding associated cardiovascular risk. In this paper the links between obesity and OSA are discussed, and the recent studies evaluating the behavioural, pharmacological and surgical approaches to weight loss in OSA are reviewed.

## 1. Introduction

Obstructive sleep apnoea (OSA) syndrome is common with a prevalence of approximately 4% in middle-aged men and 2% in middle-aged women [1]. Frequent partial (hypopnoea) or complete (apnoea) closure of the upper airway during sleep leads to oxygen desaturation, increased respiratory effort, arousal, and sleep fragmentation. Patients typically present with witnessed apnoeas, loud intermittent snoring, and excessive daytime somnolence [2]. The syndrome is associated with impairment in quality of life [3], cognitive functioning, and work performance [4], and with an increased risk of road-traffic accidents [5]. OSA is considered an independent risk factor for hypertension [6, 7] and has associations with coronary artery disease [8], stroke [9], heart failure [10], arrhythmias [11], metabolic syndrome [12], and type 2 diabetes [13]. Obesity is an important risk factor for the development of OSA [14–16] and is unique amongst the major risk factors in being modifiable [17]. There is a wealth of studies evaluating the effects of weight loss, achieved by behavioural, pharmacological, and surgical approaches, in the management of OSA in the obese patients. In this review, we will discuss the links between excess body weight and development of OSA and the different methods of achieving weight loss and their effectiveness.

## 2. Obesity as a Risk Factor for OSA

The evidence to support the role of excess weight as a causal factor in the aetiology of OSA is convincing. In a population study involving 2148, prevalence of obesity was significantly higher in those with OSA than those without, whether male (22% versus 8%) or female (32% versus 18%) [18]. Another study of 161 obese patients (BMI ≥ 30 kg/m^2^) showed that OSA was present in over 50%, and in 25% this was severe [19]. Amongst the morbidly obese patients (BMI ≥ 40 kg/m^2^), prevalence of OSA as high as 98% has been reported [20]. Using data from the population-based Wisconsin Sleep Cohort Study [1], Young et al. estimated that, in 41% of adults with mild or worse sleep disordered breathing (SDB) (AHI ≥ 5) and in 58% of those with moderate or worse SDB (AHI ≥ 15), sleep disordered breathing (SDB) was attributable to excess weight (defined as BMI ≥ 25 kg/m^2^) [16]. In a study based on data from the 2005 National Sleep Foundation Sleep in America poll, 59% of 379 obese individuals were at high risk for OSA as defined by the Berlin Questionnaire [21]. In the Sleep Heart Health Study based on 5615 adults, the odds ratio for an AHI of 15 or greater with a BMI difference of 10 kg/m^2^ was 2.4 [22]. A longitudinal population-based study of 690 adults followed up at 4 years demonstrated that a 10% weight gain predicted a 32% (95% confidence interval (CI) 20–45%) increase in AHI while a 10% weight loss predicted a 26% (95% CI 18–34%) decrease in AHI. Further, a 10% increase in weight predicted a 6-fold (95% CI 2.2–17.0) increase in the odds of developing moderate-to-severe SDB [23]. The Sleep Heart Health Study similarly confirmed progression and regression of sleep-disordered breathing (as quantified by respiratory disturbance index (RDI)) with weight gain and loss but also demonstrated that the association was stronger for males than for females [24]. Several studies have confirmed obesity and BMI [22, 25, 26] as predictors of OSA. Likewise, other relevant anthropometric measures have been associated with OSA such as neck circumference [22, 26–28], waist circumference [29], waist-hip-ratio [25], and visceral adiposity [30, 31].

## 3. Mechanisms for the Development of OSA in the Obese Patients

There are several mechanisms by which obesity could result in OSA, and these may act synergistically. It is proposed that increased peripharyngeal fat deposition results in mechanical loading that offsets the maintenance of airway patency by the dilator muscles and that this increase in collapsibility is particularly prominent during sleep when there is a reduction in neuromuscular activity [32–34]. In addition, there is some evidence to suggest that central obesity in particular may have detrimental effects on neuromuscular activity in the upper airway [35]. Obesity is associated with a reduction in functional residual capacity (FRC) [36]. Pharyngeal collapsibility may be further accentuated by this reduction in FRC and subsequent decrease in tracheal traction on the pharynx [34]. Finally, a self-perpetuating cycle may develop in which sleep disruption leads to increased appetite (especially for calorie-rich high-carbohydrate foods) [37], reduced activity levels, further weight gain, and increased severity of OSA [38].

## 4. Weight Loss as a Therapeutic Approach in OSA

### 4.1. Behavioural Methods (See Table 1)

Several studies have been performed to evaluate the effects of approaches including dietary modification and exercise, as well as counselling. Two small cohort studies [39, 40] and a larger randomised, controlled, parallel study [41] have assessed the effects of a very low-calorie diet (VLCD) given over two to three months. In each of these studies, significant improvements in weight and BMI occurred. Johansson et al. [41] reported a significant reduction in AHI with 17% “cured,” while, in the study of Kansanen et al. [40], RDI was significantly decreased with “cure” in 20%. All three studies demonstrated improvements in nocturnal oxygenation, and a reduction in daytime somnolence was reported by two [39, 41]. In a follow-on study [42], Johansson et al. enrolled patients, on the completion of the VLCD, into a weight-loss maintenance programme incorporating behaviour modification group therapy focusing on elements such as nutrition education and increased physical activity. After one year, anthropometric measures and sleep variables had increased compared to post-VLCD values but were still significantly better than at baseline, with “cure” in 10%. In another study, patients with mild-to-moderate OSA were randomised to CPAP, an oral appliance or conservative measures (sleep hygiene), and overweight individuals (BMI ≥ 23 kg/m^2^) were invited to attend a weight control programme in the local Dietetics Unit [43]. Weight loss occurred in 45 participants across the three treatment groups after 10 weeks (75.8 (1.6) kg to 72.5 (1.5) kg), and this was accompanied by a decrease in AHI (24.6 (1.7) to 19.1 (2.0)). 8 participants were cured (AHI < 5) with a mean weight loss of 2.9 (1.0) kg. In addition, there was a linear relationship between change in AHI and body weight (*r* = 0.298,  *P* = 0.004) independent of treatment modality. Kemppainen et al. performed a prospective, and randomised, controlled parallel study comparing a VLCD and supervised lifestyle intervention with routine lifestyle counselling over 3 months [44]. BMI decreased by 5.4 kg/m^2^ in the intervention group compared to only 0.49 kg/m^2^ in the control group (*P* < 0.001). The reduction in AHI was not significantly different between groups, however (intervention group: 3.2 events/hour, control group: 1.3 events/hour). Likewise, a small cohort study in which patients completed a 16-week program including VLCD and an exercise program showed significant weight loss and reduction in daytime sleepiness but no significant change in AHI after 16 weeks [45]. In this study, at one year, patients had again regained weight although weight was still significantly less than at baseline. In a randomised and controlled, parallel study, Tuomilehto et al. reported improvements following a VLCD for 12 weeks with supervised lifestyle counselling focussing on diet, exercise and lifestyle modification [46]. After one-year significant improvements were seen in AHI, number of patients cured, nocturnal oxygenation, weight, and BMI in the intervention group. Nerfeldt et al. [47] have recently reported the results of a 2-year weight reduction program that included an 8-week low-calorie diet and behavioural change support. Disappointingly, no change in AHI was found; however, there were significant reductions in BMI, ODI, arousal index, and ESS. Promising results have been reported for a program including formal cognitive behavioural therapy [48] and one including a 6-month exercise training program [49].

Thus, the outcomes of behavioural therapy for weight loss in OSA are mixed. Furthermore, for these programmes to be available in the clinical setting may not be economically feasible due to the costs involved such as employment of dietician, physiotherapist, and nurse. Moreover, drop-out rate is not insubstantial; in the study by Nerfeldt et al. 30% of patients did not complete the two-year program [47]. Maintenance of the weight loss achieved is variable particularly if long-term followup is not provided [50]. For lifestyle changes such as integration of regular exercise into the routine to be sustained is heavily dependent on individual motivation [51].

### 4.2. Pharmacological Methods (See Table 1)

Sibutramine is a serotonin and noradrenaline reuptake inhibitor that promotes weight loss by enhancing satiety and increasing energy expenditure through thermogenesis [52]. A randomised, placebo controlled trial of sibutramine, given to overweight males with OSAHS, found no change in AHI or weight over one month [53]. More recently, the results of a cohort study, in which obese males with OSAS were enrolled into a 6-month sibutramine-assisted weight loss program incorporating a dietary prescription and advice on exercise, have been reported [54, 55]. Significant improvements were seen in weight (−8.3 ± 4.7 kg), RDI (−16.3 ± 19.4 events/hr), and ESS (−4.5 ± 4.6), and 4/87 (5%) were “cured” with RDI < 5 events/hr by 6 months [54]. Significant, though modest, improvements in insulin sensitivity and lipid profile were also reported [55]. In a further study, sibutramine-assisted weight loss was compared with conventional CPAP treatment and lifestyle recommendations over one year in a nonrandomised, parallel study in obese patients with OSAS [56]. Weight decreased by 5 kg in the sibutramine treated group while remaining unchanged in the CPAP group. Sibutramine treatment was associated with no change in AHI or in ESS but an improvement in mean nocturnal oxygen saturations and also an improvement in sleep architecture. CPAP on the other hand significantly improved both AHI and ESS, led to greater improvement in sleep architecture, and led to improvements in other sleep and respiratory variables. Overall, sibutramine-assisted weight loss was concluded to be inferior to CPAP. More recently, in January 2010, the marketing authorisation for sibutramine has been suspended by the Medicines and Healthcare products Regulatory Agency due to concerns that cardiovascular risks outweighed benefits and that there may be an increased risk of nonfatal myocardial infarction and stroke [57, 58]. Orlistat is another therapeutic option for management of weight loss and this agent inhibits gastrointestinal lipase thus reducing fat absorption [59]. To the authors' knowledge no trial has evaluated the use of orlistat in management of obese sleep apnoea patients.

### 4.3. Surgical Methods (See Table 2)

The National Institute for Health and Clinical Excellence Guidelines state that bariatric surgery should be recommended as a treatment option for adults with BMI of 40 kg/m^2^ or more, or between 35 and 39.9 kg/m^2^ in the presence of significant comorbidities such as type 2 diabetes or hypertension. In addition, all nonsurgical measures should have been employed with failure to achieve or maintain adequate weight loss over at least 6 months. Further, individuals should be managed within a specialist obesity service, be both fit for anaesthesia and surgery, and willing to commit to long-term followup [57]. There are several surgical procedures that can be performed including laparoscopic adjustable gastric banding (LAGB), Roux-en-Y gastric bypass (RYGB), laparoscopic sleeve gastrectomy (LSG), and laparoscopic biliopancreatic diversion. Of these, the LAGB and RYGB are more commonly available in the UK [60]. The mechanisms by which bariatric procedures lead to weight reduction incorporate gastric restriction and/or malabsorption and can be summarised as the BRAVE effects: bile flow alteration, restriction of gastric size, anatomical gut rearrangement and altered flow of nutrients, vagal manipulation, and enteric gut hormone modulation [61]. The LAGB procedure entails placement of a band around the proximal stomach forming a pouch with a narrow outlet just distal to the gastrooesophageal junction. The degree of restriction can be altered by inflating or deflating a balloon within the band with saline via a subcutaneous port [62, 63]. In RYGB surgery, the proximal stomach is transected forming a small gastric pouch which is joined to the roux limb of jejunum with the result that the more distal stomach, the complete duodenum, and proximal jejunum are bypassed [62].

Since the 1980's, the results of several studies of the effects of surgical weight loss in patients with OSA have been published (see Table 2). To the authors' knowledge, with the exception of one [64], these have all been cohort studies either of obese individuals [65–68], specifically those with OSA [63, 69–80] or, in one case, those with respiratory comorbidity (chronic obstructive pulmonary disease, OSA, or obesity hypoventilation syndrome) requiring noninvasive positive pressure ventilation [81]. In all studies, where measured, a significant improvement in AHI occurred after surgery, in addition to the expected reduction in BMI. Furthermore, resolution of OSA occurred in up to 80% [72] however, this was variable; in the study of Lankford et al. [70], “cure” did not occur in any patient. Postoperative evaluation was carried out between 3 [72] and 28 months [71]. Other benefits have also been reported. An improvement in nocturnal oxygenation is seen with reduced oxygen desaturation index [63, 79] and time with saturation less than 90% [79, 81], increase in minimum oxygen saturation [63, 66, 69, 71, 73, 74, 78, 79] and mean oxygen saturation [63, 71, 78, 79]. Daytime hypoxaemia [80, 81] and hypercapnia [81], too, are improved. Sleep efficiency is increased [63, 69, 74, 77] and sleep architecture enhanced with decreases in stages 1 [76] and 2 [76, 77], increases in stages 3 [63, 75–77, 82] and 4 [75–77, 82], reduced REM latency [69, 74, 77], and increased REM sleep [63, 75–77, 82]. CPAP use [69, 73, 75, 78, 81] and CPAP pressure requirements [69–71, 73] are less. Likewise, there is a reduction in daytime sleepiness (ESS) [69, 73–75, 80, 83], snoring [63, 83], witnessed apnoeas [83], and other OSA-related symptoms [83] and improvement in both quality of life and depression scores [75]. One study demonstrates that spirometric measures increase significantly one year after surgery [81], and similar results were reported 6 months after intragastric balloon insertion in another study [80]. Finally, reduction in glucose, HbA1c, triglycerides, and insulin with increase in HDL have been reported with an overall reduction in metabolic syndrome [75].

From these reports, it is tempting to conclude that surgical weight loss is the panacea for OSA at least in the obese population. Caution is required in the interpretation of these studies, however, the main criticisms being that they did not include a control group, and in many cases data collection was retrospectively carried out. A large multicentre prospective controlled study with 2-year followup was carried out in Sweden by Grunstein et al. [64]. For ethical reasons, participants were not randomised and the study was questionnaire based. 1592 obese individuals undergoing various bariatric procedures were compared with 1431 matched controls that were provided with routine obesity management including dietary advice, physical training, low-calorie diets and behaviour modification. The odds ratios for development of new symptoms of apnoea, snoring, and daytime sleepiness were 0.28, 0.18, and 0.66, respectively, for the surgical group compared to the control group. Likewise, persistence of these symptoms was markedly lessened in the surgical group (approximately 20–30%) compared to controls (approximately 50–70%). Thus, surgery was associated with a marked reduction in OSA-related symptoms. Unfortunately no attempt was made to objectively measure sleep disordered breathing in this study at baseline or followup.

In summary, the aforementioned studies report promising results regarding the effects of bariatric surgery on sleep apnoea symptoms and polysomnography at least in the short term up to one to two years. What of the long term are benefits maintained? One study of LAGB surgery for obesity showed progressive loss of weight over the first 2 to 3 years with a plateau in BMI to 6 years [83], similar results being found in another study of 157 obese patients over a 5-year period [84]. This finding is at odds with the results of another study in which significant weight gain was found over a 10-year period following RYGB surgery, the increase being more in the super-obese (BMI ≥ 50 kg/m^2^) than in the morbidly obese (BMI < 50 kg/m^2^) [85]. In a study of 14 obese subjects with OSA, BMI decreased significantly from baseline (45 kg/m^2^) to 4.5 months postop (33 kg/m^2^) and increased only insignificantly (35 kg/m^2^) at 7.5 years. However, while AHI decreased significantly from 40/hr to 11/hr at 4.5 months a twofold increase to 24/hr occurred at 7.5 years, the change in AHI at 7.5 years was independent of change in BMI [76]. Another older study documented substantial weight gain and relapse of OSA in a small subgroup of obese sleep apnoea patients for which data was available at 7 years [77]. Thus, the results are conflicting but overall would suggest that weight loss is not maintained and that OSA may relapse in the years following surgery, perhaps due to factors other than just weight gain.

The morbidity and mortality related to surgical intervention should also be considered in the overall evaluation of the benefits of bariatric surgery. Grunstein et al. reported a perioperative mortality rate of 0.21% and an incidence of other complications (including bleeding, thromboembolism, wound complications, deep infections, pulmonary, and other complications) of 13% [64]. Omana et al. reported no mortalities or major complications and a rate of 15% for minor complications for LABG in 74 subjects [68]. Complications specific to LAGB surgery include band slippage or erosion, pouch enlargement, oesophageal dilatation, and oesophageal or gastric perforation while those for RYGB include staple line disruption and leak, stricture, small bowel obstruction and hernia (both internal and incisional) [86]. A large meta-analysis of mortality after bariatric surgery reported up to 30-day mortality of 0.28% and >30-day to 2-year mortality of 0.35% [87]. Concerns have been raised over the use of risk of bowel distension and subsequent anastomotic leak in association with the use of bilevel positive airway pressure in the immediate postop period after gastric bypass; however, this seems rare complication [88].

## 5. Mechanisms for the Resolution of OSA after Weight Loss

The mechanisms by which weight loss results in a reduction in severity of OSA or even resolution have been explored in a number of studies. Following weight loss, there is a reduction in nasopharyngeal collapsibility and resistance implying that the calibre of the upper airway increases [39, 89]. In the study of Busetto et al. discussed earlier, acoustic pharyngometry was utilised to measure pharyngeal cross-sectional area [80]. At baseline, pharyngeal cross-sectional areas were significantly reduced in obese individuals with OSAS compared to nonobese controls. Six months after intragastric balloon insertion, weight loss was associated with significant increases in cross sectional area at the oropharyngeal junction both upright and supine and the mean pharyngeal cross-sectional upright. However, mean pharyngeal cross-sectional area and crosssectional area at the glottis level were still significantly lower than for nonobese controls. Pharyngeal and glottic function appears to be improved [90]. The level to which the upper airway critical pressure (i.e., the nasal pressure below which inspiratory airflow ceases) falls following weight loss determines whether there is complete resolution of OSA or not [91]. The improvements seen in pharyngeal function may be related to reductions in mechanical loading particularly due to parapharyngeal fat pads. One study using CT imaging showed that velopharyngeal volume and lateral diameter are increased while facial and abdominal fat volumes decrease along with parapharyngeal fat pad volume [17]. In this study, reduction in upper airway length and in visceral abdominal fat best explained improvement in AHI after weight loss (*R* ^2^ 0.31,  *P* = 0.004), and, interestingly, changes in parapharyngeal fat did not correlate with changes in AHI. An alternative explanation is that reduction in central adiposity and the resulting reduction in production of adipokines that act on the central nervous system may lead to enhanced neuromuscular control of pharyngeal calibre [35]. Weight loss is associated with significant improvement in vital capacity, total lung volume, functional residual capacity, and forced expiratory volume [92], and this increase in lung volumes may result in increased tracheal traction on the pharynx. In the study discussed earlier by Kemppainen et al., no significant differences were found in nasal resistance or nasal volume after successful weight loss indicating that the improvement in OSA is not related to changes in nasal airflow [44]. Absence of attenuation of OSA after weight loss may be related to presence of concomitant otorhinolaryngoiatric pathology [93].

## 6. Conclusion

Obesity is a major (and perhaps the leading) risk factor for obstructive sleep apnoea. The prevalence of OSA is increased in the obese patients and vice versa; OSA is related to various anthropometric measures; the severity of OSA increases in association with weight increase. Pathophysiological mechanisms by which obesity can lead to OSA have been identified. It follows that weight loss may lead to an improvement in the severity of OSA and perhaps even it's resolution. This has been borne out in studies evaluating behavioural and surgical approaches to weight loss. Behavioural methods have focussed on dietary intervention, encouragement of exercise, and support in lifestyle change, and randomised studies have been encouraging. The evidence for benefits in association with bariatric surgery stems from cohort studies, many of them retrospective, but nonetheless persuasive. However, surgical intervention is recommended only after nonsurgical measures have failed and is associated with an albeit low mortality rate and also significant morbidity.

Although the studies of both behavioural and surgical interventions aimed at weight loss in the management of OSA have shown promising results, there are some limitations to these studies that should be noted. The majority of studies reviewed were uncontrolled and involved low subject numbers often with a male predominance. There were varying criteria for inclusion in studies with respect to the severity of obesity and both the presence and severity of OSA; the inclusion criteria for some studies included the presence of excessive daytime somnolence or other obesity-related comorbidities while other studies did not. Likewise, different methodologies were utilised for the confirmation of OSA such as polysomnography, limited sleep studies, and respiratory polygraphy, and there was variation in the outcome measures reported such as apnoea hypopnoea index, respiratory disturbance index, oxygen desaturation index, and other measures of nocturnal oxygenation. For these reasons, it is difficult to compare the different studies and to extrapolate to the wider population. The proportion of subjects lost to followup was often significant and this may lead to bias with overestimation of the improvement associated with the intervention as those not benefitting are more likely not to return for reassessment. The time to reevaluation varied from as little as 2 months to over 24 months. The benefits reported in studies with shorter durations may have been influenced by short-term simultaneous behavioural changes such as reduction of alcohol intake or increase in exercise which may not be maintained in the longer time. Alternatively shorter studies may have underestimated the potential for weight loss and attendant improvements in OSA that may be possible with longer behavioural interventions.

For either approach, behavioural or surgical, to be successful, weight loss has to be maintained in the long term to prevent relapse, and “maintenance of weight loss programmes” necessarily incorporating similar features to the behavioural weight loss programmes may not be economically feasible. Furthermore, success of such programmes or of maintenance of weight loss independently is heavily dependent on the motivation of the individual. Further randomised, controlled trials are required to confirm the beneficial effects of bariatric surgery, and those of the behavioural interventions. Studies are necessary to identify those in which behavioural therapy is likely to be effective so that limited resources are efficiently utilised.

## Figures and Tables

**Table 1:** A summary of studies of behaviourally and pharmacologically induced weight loss in obstructive sleep apnoea.

Paper	Design	*n*	% Male	Group	Intervention	Followup months	ΔBMI	ΔAHI	% With OSA cure
Johansson et al., 2011 [42]	Prospective cohort	63	100	BMI 30–40, AHI ≥ 15	VLCD and weight maintenance programme	12	35→31	36→19	10
Nerfeldt et al., 2010 [47]	Prospective cohort	33	73%	BMI ≥ 30, AHI ≥ 10 and/or ODI ≥ 6, OSAS symptoms	LCD and behavioural change support	24	40→35	43→28	?
Johansson et al., 2009 [41]	Randomised, controlled, and parallel group	63	100	BMI 30–40, AHI ≥ 15	VLCD	2	34→29 v 35→35	37→12 v 37→35	17 v 0
Tuomilehto et al., 2009 [46]	Randomised, controlled, and parallel group	72	74	BMI 28–40 kg/m^2^, AHI 5–15 events/hr	VLCD, supervised lifestyle counselling	12	33→? v 31→?	10→6 v 9→10	63% v 35%
Barnes et al., 2009 [45]	Cohort	12	25	BMI > 30 kg/m^2^, AHI 10–50 events/hr	VLCD and exercise programme	12	36→30	25→18	0
Foster et al., 2009 [94]	Randomised, controlled, and parallel group	264	41	BMI ≥ 25 kg/m^2^, AHI ≥ 5 events/hr, type 2 diabetes	Intensive lifestyle intervention (diet/exercise)	12	37→? v 37→?	23→18 v 24→28	14 v 4
Kemppainen et al., 2008 [44]	Randomised, controlled, and parallel group	52	79	BMI 28–40 kg/m^2^, AHI 5–15 events/hr	VLCD, supervised lifestyle counselling	3	33→? v 32→?	11→8 v 9→8	?
Kajaste et al., 2004 [48]	Cohort	31	100	BMI > 35 kg/m^2^, ODI > 10 events/hr	VLCD, CBT weight reduction program	24	44→40	51→32 (ODI)	?
Kansanen et al., 1998 [40]	Prospective, cohort	15	93	Overweight with OSA	VLCD	3	38→35	31→19 (ODI)	20
Suratt et al., 1992 [39]	Cohort	8	62	Obese with OSA	VLCD	?	54→46	90→62	?
Ferland et al., 2009 [56]	Nonrandomised, parallel group	40	88	BMI ≥ 30 kg/m^2^, OSAS	Sibutramine, diet and exercise v CPAP	12	37→35 v 36→36	40→37 v 44→4	?
Phillips et al., 2009 [55]	Cohort	93	100	BMI 30–38 kg/m^2^, RDI ≥ 15 events/hr	Sibutramine, diet and exercise	6	34→32	46→30 (RDI)	?
Yee et al., 2007 [54]	Cohort	87	100	BMI 30–38 kg/m^2^, RDI ≥ 15 events/hr	Sibutramine, diet and exercise	6	34→32	46→30 (RDI)	5
Martinez and Basile, 2005 [53]	Randomised, double-blind, and controlled group	19	100	BMI 25–35 kg/m^2^, AHI ≥ 10 events/hr	Sibutramine v placebo	1	?	28→27 v 28→28	?

Abbreviations: ΔAHI: apnoea hypopnoea index before and after intervention, ΔBMI: body mass index before and after intervention, CBT: cognitive behavioural therapy, CPAP: continuous positive airway pressure, *n* number, ODI: oxygen desaturation index, OSA: obstructive sleep apnoea, OSAS: obstructive sleep apnoea syndrome, RDI: respiratory disturbance index, (V)LCD: (very) low calorie diet.

**Table 2:** A summary of studies of surgically induced weight loss in obstructive sleep apnoea.

Paper	Design	*n*	% Male	Group	Procedure	Followup months	ΔBMI	ΔAHI	% with OSA cure
Behrens et al., 2011 [65]	Retrospective, cohort	34 (21 OSA)	3	BMI > 30	LSG	10	50.3→39.9	?	76
Martí-Valeri et al., 2007 [81]	Cohort	30 (14 OSA)	90	Obese with associated respiratory comorbidity requiring NIPPV	RYGB	12	56→32	64→17 (RDI)	?
Fritscher et al., 2007 [78]	Cohort	12	75	BMI ≥ 35 with obesity-related comorbidity or ≥40 without and AHI ≥ 15	RYGB	18	56→34	46→16	25
Haines et al., 2007 [69]	Prospective, cohort	101	?	Obese, RDI > 5, ESS ≥ 6	RYGB	11	56→38	51→15 (RDI)	?
Kalra et al., 2005 [66]	Retrospective, cohort	34 (19 AHI ≥ 5)	?	Adolescent, BMI ≥ 40, obesity-related comorbidity	RYGB	5	60.8→41.6	9.1→0.6	?
Lankford et al., 2005 [70]	Retrospective, cohort	15	40	Obese, OSA	RYGB	12	48→32	40→?	0
Guardiano et al., 2003 [71]	Retrospective, cohort	8	12	Obese, OSA	RYGB	28	49→34	55→14 (RDI)	50
Peiser et al., 1984 [72]	Cohort	15	93	Morbidly obese with OSA	RYGB	3	?	82→15	80
Lettieri et al., 2008 [73]	Retrospective, cohort	24	25	Obese with EDS, AHI ≥ 5	GB	12	51→32	48→24	4
Rasheid et al., 2003 [74]	Prospective, cohort	11	?	Obese, ESS ≥ 6, RDI > 5	GB	12	62→40	56→23 (RDI)	?
Rao et al., 2009 [63]	Retrospective, cohort	46	?	BMI ≥ 32.5 with obesity-related comorbidity or ≥37.5 without and AHI ≥ 15	LAGB	13	45→30	38→13	78
Dixon et al., 2005 [75]	Prospective, cohort	25	68	BMI > 35, AHI > 25	LAGB	18	53→37	62→13	?
Busetto et al., 2005 [80]	Cohort	17	100	BMI > 50, AHI > 20	IGB	6	56→49	59→14	59
Grunstein et al., 2007 [64]	Prospective, controlled, nonrandomised	3023	30	Female: BMI ≥ 38, male: BMI ≥ 34	Various	24	42→32	?	?
Valencia-Flores et al., 2004 [67]	Prospective, cohort	29	45	Morbidly obese	Various	14	56→39	52→?	46
Poitou et al., 2006 [79]	Prospective cohort	35	17	BMI > 40, AHI > 10	RYGB, LAGB	12	51→40	24→10	63
Pillar et al., 1994 [76]	Cohort	14	79	Morbidly obese with OSA	RYGB, VBG	4	45→33	40→11	43
Charuzi et al., 1992 [77]	Cohort	47	?	Morbidly obese with OSA	RYGB, VBG	10	?	61→8	40
Omana et al., 2010 [68]	Retrospective, cohorts	123	24	Obese	LSG (49) v LAGB (74)	15 17	52→? 44→?	? ?	55 25

Abbreviations: ΔAHI: apnoea hypopnoea index before and after intervention, ΔBMI: body mass index before and after intervention, EDS: excessive daytime somnolence, ESS: epworth sleepiness scale, GB: gastric bypass, IGB: intragastric balloon, LAGB: laparoscopic adjustable gastric banding, LSG: laparoscopic sleeve gastrectomy, *n* number, NIPPV: noninvasive positive pressure ventilation, OSA: obstructive sleep apnoea, RDI: respiratory disturbance index, RYGB: roux-en-Y gastric bypass, VBG: vertical banded.
